# Interpersonal Relationship, Knowledge Characteristic, and Knowledge Sharing Behavior of Online Community Members: A TAM Perspective

**DOI:** 10.1155/2022/4188480

**Published:** 2022-10-10

**Authors:** Wu Jiarui, Zhang Xiaoli, Su Jiafu

**Affiliations:** ^1^Hotel Management Institute, Yunnan Tourism College, Kunming 650221, China; ^2^Anhui Technical College of Mechanical and Electrical Engineering, Wuhu 241000, China; ^3^Chongqing City Vocational College, Chongqing 402160, China; ^4^International College, Krirk University, Bangkok 10220, Thailand; ^5^National Research Base of Intelligent Manufacturing Service, Chongqing Technology and Business University, Chongqing 400067, China

## Abstract

With the rapid development of information science and technology, online communities are attracting an increasing number of participants, who can share information, create original content, and offer emotional support, thus communicating and spreading knowledge frequently within the community. To develop a model of influencing factors for the knowledge sharing behavior of online community members, this study employs the technology acceptance model (TAM) as a moderator variable based on the social exchange theory. In this study, the influencing factors model for knowledge sharing behavior of online community members was tested using PLS-SEM. The results show that knowledge sharing is motivated by trust and quality of knowledge; the interaction term of perceived usefulness and knowledge quality of the user has a significant negative correlation with the knowledge sharing behavior of online community users; perceived usefulness significantly positively moderates the correlation between knowledge tacitness and knowledge sharing behavior of users; perceived ease of use significantly positively moderates the relationship between knowledge quality and knowledge sharing behavior; perceived ease of use significantly negatively moderates the relationship between knowledge tacit and knowledge sharing behavior. In order to maximize the activity and stickiness of the online community platform, the platform must focus on maintaining and enhancing the platform's credibility and knowledge quality. On the other hand, the online community platform extols its professional utility and ease of operation, which are conducive to the generation of behavior that is conducive to knowledge sharing.

## 1. Introduction

Following the vigorous spread of Internet technology and Web2.0, the influence of the Internet on the life and work of people is growing with each passing day [1]. The online community based on social media has gradually turned into a new platform for people to share knowledge and publish different ideas, and it also could facilitate business activities. The social network has the potential to change the way people live and facilitate knowledge exchange among individuals. The site contains a number of groups created by users with similar hobbies and interests who share their views, experiences, and insights on a variety of topics and seek out knowledge and information related to their interests [2]. For instance, the Facebook group is quite different from the traditional social networking for people. The platform facilitates communication between users with common interests, discusses, and shares information. As for another example, Zhihu is a respected and well-known Q&A platform in the Chinese Internet. It is committed to building a knowledge sharing network that will be accessible to everyone, allowing people to easily share their knowledge, experience, and insights worldwide.

The online community is a product of rapid growth and has a huge influence in the Internet era. Lai and Chen [3] illustrate that an online community consists of a group of people with common interests who interact virtually one-on-one. Additionally, they can assist with the retention of customers, word-of-mouth advertising, product reviews, and customer insight. Online communities are critical for continuing commitment to interact, the relationship of trust, and meaningful interactions with partners, customers, employees, and suppliers [3]. Members could through these communities seek and share knowledge [4]. Xu and Liu [5] demonstrate that due to its characteristics of fairness, openness, cross-time and spatiality, it has the ability to integrate dispersed knowledge groups on a larger scale, even globally. As a result of the growth in online communities over the past few years, online communities have the potential to play a significant role in enhancing the exchange of knowledge.

Recently, scholars [6–9] have conducted some studies on user information sharing behavior in online communities. Gang [6] studied the influence of the motivation of professional virtual community members to participate in knowledge exchange on interactive behavior. Connelly et al. [7] revealed that people may not share knowledge because they are unaware of the needs of knowledge of others. Li et al. [8] detected that online community members' knowledge sharing intentions were significantly affected by altruism, relational trust, personal outcome expectations, and self-efficacy. Sun and Hong [9] showed that human interaction, human interaction with information and expectations of the outcome, expectations of the community, and user knowledge sharing behaviors were significantly positively correlated, and the interaction between people and systems was positively affected by user knowledge sharing behaviors.

The previous research studies bulkily stayed on the single level of human interaction, human-knowledge interaction, or human-system interaction; nevertheless, there is a lack of integrative study. At first, people as the subject of knowledge sharing are the bearers and initiators of knowledge sharing activities. It is likely that the subjective initiative of individuals will affect the judgment of objective phenomena. Such as, emotions have a profound effect on people's willingness to share information. Second, knowledge is composed of explicit and implicit, among which tacit knowledge accounts for a larger proportion than explicit knowledge. This is a state of being that exists in people's minds, which is difficult to describe adequately in language or in written materials, and is not easy to be comprehended by others. Finally, since information network technology is an important tool for knowledge sharing to be carried out smoothly; whereas, in different industries, the use of multiple systems is independent and incompatible with each other, which reduces the effectiveness of knowledge sharing. Therefore, by integrating human interaction, human-knowledge interaction, and human-system interaction, the analysis results are objective and scientific.

This study selects two dimensions of interpersonal relationships (reciprocity and trust) and knowledge characteristics (quality of knowledge and tacit knowledge) as research objects, according to the theory of technology acceptance, the introduction of perceived usefulness and perceived ease of use has a moderating effect on interpersonal relationships and knowledge sharing behaviors, as well as knowledge characteristics and knowledge sharing behaviors. This is of great significance for the online community to remain the willingness of users to participate, maintain the vitality of the community, and promote the active participation of community members in knowledge sharing.

Accordingly, the remainder of the study is organized as follows: Section 2 investigates the influences of interpersonal relationships, knowledge characteristics, and technology acceptance models on knowledge sharing behavior. In Section 3, an assessment of the impact the technology acceptance model has had on the behavior of knowledge sharing is made.  Section 4 assesses how knowledge sharing behaviors are influenced by moderator variables.  Section 5 concludes the study and discusses and summarizes the contributions and limitations.

## 2. Literature Review and Hypotheses Development

### 2.1. Interpersonal Relationships and Knowledge Sharing Behavior

It is imperative that interpersonal communication can be practiced in modern society. For one to truly appreciate others, he or she must understand their needs and desires. Primarily, a mutually beneficial relationship is one in which both parties expect some kind of gain, whether it is a material gain or an emotional one. The effectiveness of interpersonal interactions depends on how beneficial they are for both parties, as a relationship cannot last if one party is always benefited alone. Second, trust is the cornerstone of any relationship between two individuals. A harmonious interpersonal relationship cannot exist without trust as the basis of words and deeds. The term trust refers to the subjective sense of confidence and security that one party has toward the other party, which is often associated with the cooperative behavior of people and the willingness to act [10]. As variables in the social exchange theory, reciprocity and trust can be used to explain models of behavior involving knowledge sharing. Blau[11] elucidated that the social exchange theory has been employed to explain knowledge sharing behavior in terms of people's behavior, outcomes or interests, and the environment. Therefore, this study investigates the effect of reciprocity and trust, two interpersonal factors, on knowledge sharing behavior, based on the social theory as well as social exchange and social cognition theories [12].

#### 2.1.1. Reciprocity and Knowledge Sharing

As an extrinsic motivation, reciprocity suggests that people will exhibit knowledge sharing behaviors with the aim of accumulating rewards. The concept of knowledge sharing in the early years was defined by Polanyi [12] as a deliberate subjective behavior that can be reused by others through knowledge transfer. As per Okyere-Kwakye [13], individuals may not share initially, since they perceive the activity as a mere cost, unless they intend to share when a positive reward is expected. Using social exchange theory as a basis for reciprocity, it is suggested that individuals only engage in certain behaviors if they expect a positive outcome [11]. It has been found by Kelley and Thibaut [14] that participants in online communities share their individual knowledge when they perceive the behavior of other participants as being similar to their own. Among participants in a community of practice (CoP), Wasko and Faraj [15] demonstrated that reciprocal behaviors enhance knowledge sharing. Thus, the first hypothesis is proposed.

H1: knowledge sharing behavior is positively influenced by reciprocity.

#### 2.1.2. Trust and Knowledge Sharing

It is believed that trust is an intrinsic motivation to act on behalf of people based on an appreciation for the benefits of their actions and is the focal point of all relationships within an organization [10, 16]. As Molm [17] asserted, individuals refrain from engaging in certain activities when they are uncertain of the future rewards associated with them. The result is that people act according to how much trust they have in the system, but they can only build trust in others if they are confident, and there will be no costs associated with transactions [17]. A trusting relationship between two people facilitates easy cooperation. Nevertheless, Molm [17] also noted that trust encourages members of an organization to share knowledge. There will be a trend towards higher cooperation and commitment as long as trust exists among individuals in an organization. Some literatures contend that trust is the most cost-effective technique for enhancing knowledge sharing within an organization [18]. According to this hypothesis, participants are more likely to share knowledge with a recipient if they perceive the recipient to be honest, trustworthy, and reliable. A higher level of trust will prevent the individual from thinking of any future negative things at the event and will encourage a willingness to share knowledge. Thus, the next hypothesis can be formulated.

H2: knowledge sharing behavior is positively influenced by the trust.

### 2.2. Knowledge Characteristics and Knowledge Sharing

On account of communities are often the basic social units that organize knowledge creation, the study of knowledge sharing in community settings becomes increasingly important [19, 20]. Furthermore, knowledge sharing demands collaboration on the part of both the seekers and contributors [21], as knowledge sharing can be facilitated while knowledge can be exchanged synchronously and asynchronously [22]. Yan and Jian [23] mentioned that as a commodity, knowledge has its natural attributes (use value) and its social attributes (value). The choice of knowledge commodities by consumers is not only solely dependent on their own perception of the quality of content but also upon the source from which the knowledge was derived. A related study on the knowledge acquisition behavior of users shows that high-quality knowledge promotes users to use knowledge communities.

#### 2.2.1. Knowledge Quality and Knowledge Sharing

Knowledge quality pertains to how well new knowledge satisfies the criteria of study in terms of productivity-enhancing properties, affecting social life, saving trial-and-error costs, and knowledge value-added [24]. The quality of knowledge referred to in this study refers to the higher knowledge quality exchanged and shared in the online community, and the greater the probability of members participating in sharing. The following assumptions are derived from this information:

H3: knowledge sharing behaviors are positively influenced by knowledge quality.

#### 2.2.2. Knowledge Tacitness and Knowledge Sharing

Yan et al. [25] referred that the exchange of information in the online community not only contains a lot of explicit knowledge but also hides a wealth of tacit knowledge. Knowledge sharing is an exchange behavior. Users will be motivated to share knowledge if they perceive the benefits that sharing knowledge can bring to them, whether that is in the form of material reward or spiritual reward [26]. Jeremy [27] explained that it is generally believed that tacit knowledge (as distinct from the more general intangible investment) cannot be codified, imperceptive knowledge of how it is acquired through informal acceptance of learned behaviors and procedures. The knowledge that is tacit is internalized within an organization, exhibits characteristics which cannot be expressed in documents, exhibits a lower degree of fluidity than explicit knowledge, and is very hard to imitate [28]. Therefore, knowledge tacitness possesses the characteristics of a valuable resource.

While knowledge itself is valuable for generating competitive advantage and value within an organization, it is not sufficient to generate those benefits on its own. The value of knowledge cannot be realized until it is shared within the organization in order to generate a competitive advantage. In light of this, it is necessary to integrate and externalize tacitness knowledge in order to gradually convert it into coded and explicit knowledge [29] because the more explicit the knowledge, the easier it will be for organizations to share it. Therefore, the next hypothesis can be made.

H4: knowledge sharing behavior is positively influenced by tacit knowledge.

### 2.3. Moderating Role of Perceived Usefulness

The technology acceptance model (TAM) proposed by Davis believes that the user's perception of human-computer interaction can be divided into perceived usefulness and perceived ease of use [30]. The term perceived usefulness is defined here as “the degree to which an individual believes that using a specific system will hoist his or her performance on the job.” This stems from the definition of the word useful: “capable of being utilized to one's benefit.” In organizational settings, people perform various rewards in order to reinforce their performance. Conversely, a system with a high perceived utility is considered to have an effective use-performance relationship by its users [30].

Recent studies have investigated the regulatory role of TAM factors. It has been demonstrated that a platform that is deemed useful and easy to use will boost influencers, thereby enhancing the satisfaction of members and increasing their behavioral intentions. Considering community participation and contribution in the context of online communities, social media are used by individuals to participate and contribute to online content for two reasons, namely, participation benefits and contribution incentives [31]. As a whole, online communities offer a number of benefits to their members. Participation benefits are structured around four basic needs, namely, needs of functional, needs of social, needs of psychological, and needs of hedonic [32]. First, users can benefit from these platforms on a functional level by joining online communities for specific activities [33]. In parallel, they will also be able to build trust and expand their online social network in the process of information exchange [34]. Thus, the following assumptions are proposed:

H5: perceived usefulness positively modulates reciprocity and knowledge sharing behaviors.

H6: perceived usefulness positively modulates trust and knowledge sharing behaviors.

H7: perceived usefulness positively modulates the quality of knowledge and knowledge sharing behaviors.

H8: perceived usefulness positively modulates tacit knowledge and knowledge sharing behaviors.

### 2.4. Moderating Role of Perceived Ease of Use

By contrast, in terms of perceived ease of use, it refers to the degree to which a user perceives a particular system as effortless. This stems from the definition of “easy” and “without difficulty or effort.” In other words, when all else are equal, users are more likely to accept an app that is considered to be easier to use than other apps [30].

In contrast, contributions to online communities can be attributed to several types of factors. Like those who join online communities for the purpose of participation, members participate in the creation of content as a means of reward. There are five motivations which can be identified with regard to participation in online communities—instrumentality, effectiveness, quality control, acquisition of status, and expectations [31]. These incentives increase not only engagement but also increase contributions, sparking interactive activity on the online platform. Parra-López et al. [35] extended a factor model that assumes that users will bear the cost of effort, spend time learning the system, and suffer privacy losses, all of which may undermine their motivation to contribute in online communities [36–38]. Accordingly, the following assumptions are made:

H9: perceived ease of use positively modulates reciprocity and knowledge sharing behaviors.

H10: perceived ease of use positively modulates trust and knowledge sharing behaviors.

H11: perceived ease of use positively modulates quality of knowledge and knowledge sharing behaviors.

H12: perceived ease of use positively modulates tacit knowledge and knowledge sharing behaviors.

**Figure 1:**
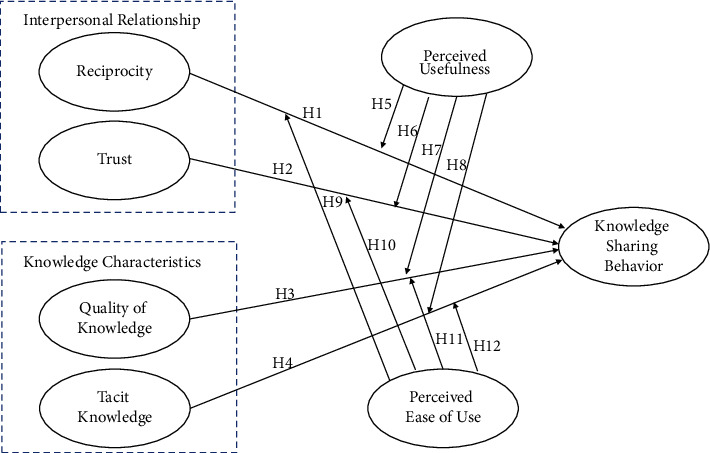
Research model.

Therefore, this study is based on the social exchange theory, combined with knowledge characteristics, and introduces a technology acceptance model as a moderator variable to develop a model of the factors that influence knowledge sharing behaviors in network communities, as shown in Figure 1.

## 3. Methodology

### 3.1. Measurement Development

There were several variables and questionnaires used in this study that were adapted from mature scales used in previous studies, and some of them were designed according to the study object and study requirements set forth in the study. There are three parts to the questionnaire: the first part is the characteristics of the demographic and the duration of using the online community; the second part is the measurement of interpersonal relationships, knowledge characteristics, and the moderator variables of the TAM model; the third part is the measurement of self-construction. All items proposed in this article were designed using a five-level Likert scale (5 means strongly agree and 1 means strongly disagree).

### 3.2. Data Collection

After completing the questionnaire design, the experts evaluated the questionnaire, reviewed the necessity and usefulness of the questionnaire items for measuring variables, confirmed that all factors were related to the corresponding variables, and assessed the correctness and appropriateness of the word and content of the questionnaire. Some wording and presentation of the questionnaire have been adjusted based on their feedback.

The questionnaire survey was conducted online, produced through the Questionnaire Star platform, and collected data by WeChat forwarding and snowballing. The collection time was from May 4th to 12th, 2022, and a total of 336 valid questionnaires have been recovered.

The features of the demographic profile of the questionnaire sample and the duration of using the online community are shown in Table 1. In terms of gender composition, males and females accounted for 43.75% and 56.25% of the respondents respectively; most of the users were between 18 and 28 years old, and most of them used online communities for 4–6 years, indicating that the respondents have extensive experience in using the online community.

### 3.3. Data Analysis

#### 3.3.1. Reliability

As a means of analyzing the reliability and validity of the collected effective data, the reliability and validity of the survey samples were evaluated. In reliability testing, the consistency, stability, and dependability of test results and data were examined. This study uses SmartLPLS3.0 to test the internal consistency coefficient (Cronbach's *α*) and composite reliability of each study variable.  Table 2 presents the results of the test. The consistency coefficient and composite reliability value of each variable in this study are above the critical value of 0.7 [39], which satisfy the reliability requirements, indicating that the measurement model has high internal consistency and reliability.

#### 3.3.2. Validity

Validity includes content validity and construct validity. It is important to note that some of the questions in the questionnaire were adapted from existing literature, while others were designed in conjunction with the characteristics of the study object, providing validity to their content. There are two primary measures of construct validity, namely, convergent validity and discriminant validity. Convergent validity reflects whether the measurement items of a variable are highly correlated. The main measurement indicators include factor loading and average variance extraction value. It is generally believed that if the factor loading is greater than 0.7 and the AVE is above 0.5, the scale is considered to have high convergent validity.  Table 2 elucidates that the standardized loadings of items were mostly higher than 0.7 and the AVE for every construct was greater than 0.5, indicating that the model has good convergent validity.

Discriminant validity reflects whether the correlation between the measurement items of different variables is as small as possible. The standard for evaluating each variable is that the square root of the AVE value must be greater than the correlation coefficient between the variable and the other variables [40]. As illustrated in Table 3, in each case, the square root of the AVE value (the value of the diagonal line) is above than the correlation coefficient between the variable and the other variables, which means that the model has excellent discriminant validity [40].

## 4. Results Analysis

### 4.1. PLS-SEM Testing

It is usually possible to use three types of methods when studying the relationship between variables. This study is based on the partial least squares method, which was proposed by World et al. in 1983 [41]. It is a new type of multivariate statistical analysis method, which incorporates both regression analysis and structural equation modeling. It is capable of constructing regression paths for multiple dependent variables and also for testing mediation and moderation effects at one time. It is suitable for small samples, and it is robust to collinearity problems and normally distributed data, and there will be no problems such as the model cannot be identified or the coefficient is greater than 1 which is caused by too many single measurement indicators, which makes up for the shortcomings of the traditional structural equation model.

### 4.2. Main Effects Test

On the premise of the reliability and validity of the measurement model, the PLS-SEM method was applied with the aid of SmartPLS 3.0 software. The bootstrap repeated sampling method was used to test the significance of the collected data, and the sampling number was 5000.

In this study, the detection model was constructed in three steps for analysis and comparison: a first assessment was made on the effects of the control variable on independent and dependent variables, then the moderator variables were added, and finally, the interaction terms between the moderator variables and their respective variables were added.

**Figure 2:**
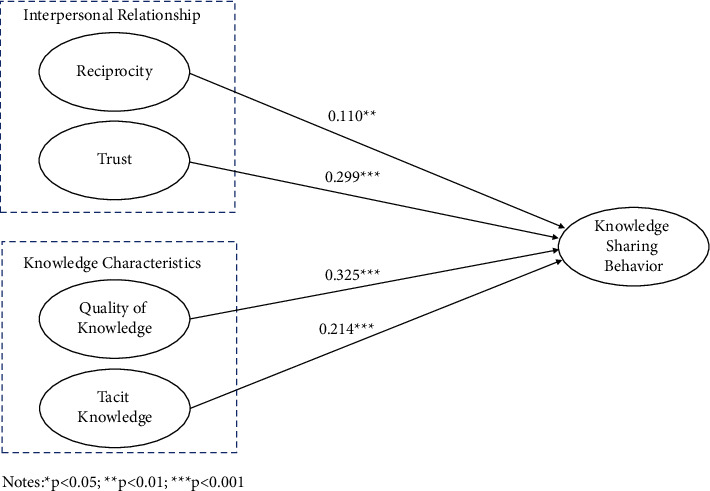
Main effects test.

In the first test (model 1), the three control variables of gender, age, and occupation have no significant correlation with knowledge sharing behavior.  Figure 2 illustrates the effects of each variable on knowledge sharing behavior.

Based on Figure 2, it can be seen that in the first dimension, reciprocity (*β* = 0.110, *p* < 0.01) and trust (*β* = 0.299, *p* < 0.001) have a significant positive impact on knowledge sharing behavior; thus, H1 and H2 were supported; second, quality of knowledge (*β* = 0.325, *p* < 0.001) and tacit knowledge (*β* = 0,214, *p* < 0.001) have a highly significant positive correlation with knowledge sharing behavior; thus, H3 and H4 were supported.

### 4.3. Moderated Variable Interaction Test

The second step of testing (model 2) introduces the moderating variable TAM model, that is, the influence of perceived usefulness and perceived ease of use on knowledge sharing. The test results reveal that perceived usefulness (*β* = 0.409, *p* < 0.001) has a significantly positive impact on knowledge sharing behavior; moreover, perceived ease of use (*β* = 0.165, *p* < 0.05) correlates positively with knowledge sharing behavior. Nevertheless, when the influence of moderator variables on knowledge sharing behavior is added, knowledge sharing behavior is not significantly affected by latent variables such as trust and tacit knowledge.

**Figure 3:**
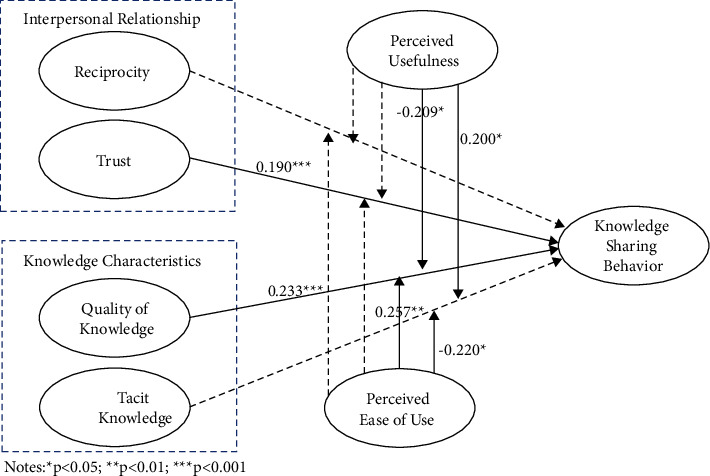
Moderated variable interaction test.

In the third step of test (model 3), in order to detect the moderating effect, the two TAM model variables are interacted with four latent variables, respectively, to form eight interaction terms, and the path coefficient and the correlation of the interaction terms to knowledge sharing behavior is detected and shown in Figure 3, and the results of each hypothesis are presented in Table 4.

Figure 3 shows that the relationship between reciprocity and knowledge sharing behaviors is not significantly moderated by perceived usefulness; thus, H5 was not supported. Meanwhile, perceived usefulness also does not have a significantly moderate relationship between trust and knowledge sharing behavior; therefore H6 was not supported. Perceived usefulness significantly negatively moderates the relationship between knowledge quality and knowledge sharing behavior (*β* = −0.209, *p* < 0.05); whereas, in the main effect, knowledge sharing behavior is significantly positively influenced by knowledge quality, indicating that for users with higher perceived usefulness, the quality of their knowledge will reduce their knowledge sharing behaviors. Thereby, H7 was not supported. Knowledge tacitness and knowledge sharing behavior are significantly positively mediated by perceived usefulness (*β* = −0.200, *p* < 0.05); thus, H8 was supported.

In the moderating effect test of perceived ease of use, knowledge sharing behavior did not significantly correlate with the interaction items formed by perceived ease of use and reciprocity and perceived ease of use and trust, indicating that neither H9 nor H10 obtained support. Nonetheless, a positive correlation of knowledge sharing behavior exists between perceived ease of use and quality of knowledge as an interaction term (*β* = −0.257, *p* < 0.01), and the correlation coefficient is greater than the path coefficient of knowledge quality and knowledge sharing behavior in model 3 (*β* = 0.233, *p* < 0.001), that is, the perceived ease of use significantly moderates the relationship between knowledge quality and knowledge sharing behavior; therefore, H11 was supported. The perceived ease of use significantly negatively moderates the relationship between tacitness knowledge and knowledge sharing behavior (*β* = −0.220, *p* < 0.05); thus, H12 was not supported, and observations reveal that users with higher perceived ease of use and knowledge tacitness will degrade their behavior towards knowledge sharing.

## 5. Conclusion

### 5.1. Discussion

Based on the data study results, this study draws the three following conclusions:

1. Trust and quality of knowledge are the main motivations for knowledge sharing behavior, based on the results of the previous study by Ridings et al. [42] and Zhang et al. [43]. Trust is the premise and foundation of knowledge sharing behavior; trust can be explained as people believe that the recipient is honest, trustworthy, and reliable, and this will motivate them to share their knowledge; hence, they will seek help from other members of the online community when they encounter problems, to ask questions, communicate, and discuss in the community and be willing to accept the experience and knowledge shared by others. Conversely, acquiring high-quality knowledge serves as an internal driving force for fostering knowledge sharing behavior, knowledge quality is the primary driving force for encouraging users to engage in community activities, and high-quality knowledge can satisfy individuals' external knowledge desires. Exchange is conducive to efficient knowledge consolidation and innovation among members and is also avail to the development and growth of online communities.
2. Furthermore, reciprocity and knowledge tacitness factors will also possess a positive impact on knowledge sharing. However, there is still considerable room for improvement. Possibly, this is due to the lack of clearly defined reciprocity norms of the online community, the lack of a strong atmosphere of reciprocity, or the degree to which individual differences are felt and the existence of an independent sense of self. Compared with explicit knowledge, tacit knowledge is difficult to describe, cannot be expressed precisely, and shows less mobility. It has strong unique characteristics, and it is difficult for individuals to share and understand. However, there is also a need to take into consideration the influence of reciprocal and demand for tacit knowledge in knowledge sharing behaviors.
3. Perceived usefulness and perceived ease of use have significant moderating effects on knowledge characteristics (quality of knowledge and tacit knowledge) in knowledge sharing behavior. From the perspective of moderating effects, the interaction items of users' perceived usefulness and knowledge quality have a significantly negative correlation with the knowledge sharing behavior of online community users. Users who perceive usefulness generally need community members to help solve problems or provide assistance, and as these problems are usually not complex and difficult to solve, members who are easy to solve and do not require a high-quality knowledge level are more willing to share. Furthermore, high-quality content sharing can increase community stickiness and create a more active community.

Perceived usefulness has a significantly positive moderating impact on the relationship between knowledge tacitness and knowledge sharing behavior. Users in a community are often in a network of relationships formed by holding the same purpose, using social networks to break communication barriers and enhance social links. If individuals in the community exchange and share experiences, everyone could usually perceive each other and actively participate in it, to exchange and share their own experiences.

There is a significant positive moderating effect of perceived ease of use on the relationship between knowledge quality and knowledge sharing behaviors. This could explain that with the growth of information technology, people can easily use technology to spread and share high-quality knowledge. The interaction terms formed by perceived ease of use and knowledge tacitness have a significantly negative correlation with knowledge sharing behavior in online communities. This is because the development of technology has caused the problem of rights protection for the sharing of personal experience.

The ability to easily acquire useful and high-quality knowledge and tacit knowledge for the online community will motivate users to actively participate. This also explains why users pay to participate in some online community knowledge sharing activities related to their profession. Improving the professional and tacit knowledge of the online community can enhance the activity and stickiness of the online community.

It was not found that perceived ease of use and perceived usefulness moderated interpersonal relationships (reciprocity and trust) in knowledge sharing behavior. In terms of perceived ease of use and perceived usefulness, the effects of reciprocity and trust on knowledge sharing behaviors did not change significantly.

### 5.2. Contribution and Limitation

The sharing of knowledge among members of a network community represents a new mode of dissemination of knowledge. This study starts from the two dimensions of interpersonal relationships (reciprocity and trust) and knowledge characteristics (knowledge quality and tacit knowledge), meanwhile introducing two dimensions of perceived usefulness and perceived ease of use as moderating variables to construct a new theoretical model. This study found that trust and quality of knowledge were the main motivations for knowledge sharing behavior, and perceived ease of use and perceived usefulness had a significant moderating effect on knowledge characteristics (quality of knowledge and tacit knowledge) in knowledge sharing behavior. In this study, a holistic study is conducted on the interaction of humans, the interaction between humans and knowledge, and the interaction between humans and systems.

Practical guiding significance: (1) the online community platform should focus on maintaining and hoisting the credibility and knowledge quality of the platform, thereby improving the activity and stickiness of the platform and (2) the online community platform exalts the professional pertinence and operation convenience of the platform. In this way, knowledge sharing behaviors are more likely to be generated.

Despite the fact that this study examines knowledge sharing behavior from the perspectives of reciprocity, trust, knowledge quality, and knowledge tacitness, there are still some limitations that remain.

This study does not analyze the differences between different genders and different groups of age levels. For example, there may be differences between males and females or there may be differences in knowledge sharing behaviors among different age groups, and these factors may need to be further studied.

## Figures and Tables

**Table 1:** Demographic characteristics of the sample and duration of using online.

Variable	Index	WeChat (*N* = 336)
Gender	Male	43.75%
	Female	56.25%

Age	<18	0.89%
	18–28	67.56%
	29–40	27.38%
	41–48	2.98%
	49–55	0.30%
	55>	0.89%

Occupation	Public officials (public officials of state organs, civil servants, and other state units)	11.31%
	Professional and technical personnel (local professional and technical personnel such as doctors, lawyers, and drivers)	11.01%
	Private enterprises (entrepreneurs, individual, industrial, and commercial households)	11.90%
	Others	65.77%

Duration of using the online community (year)	<1	0.89%
	1–3	16.07%
	4–6	79.46%
	7–10	2.68%
	>10	0.89%

**Table 2:** Scale properties.

Factor	Item	Standardized loading	Cronbach's alpha	Composite reliability (CR)	Average variance extracted (AVE)
KQ	KQ1	0.818	0.924	0.943	0.768
	KQ2	0.827			
	KQ3	0.929			
	KQ4	0.925			
	KQ5	0.878			

KSB	KSB1	0.836	0.830	0.898	0.747
	KSB2	0.890			
	KSB3	0.865			

PEOU	PEOU1	0.924	0.880	0.926	0.807
	PEOU2	0.925			
	PEOU3	0.844			

PU	PU1	0.930	0.918	0.948	0.86
	PU2	0.926			
	PU3	0.925			

RE	RE1	0.897	0.842	0.905	0.762
	RE2	0.910			
	RE3	0.808			

TK	TK1	0.872	0.772	0.866	0.684
	TK2	0.751			
	TK3	0.853			

TR	TR1	0.868	0.904	0.933	0.776
	TR2	0.885			
	TR3	0.905			
	TR4	0.864			

**Table 3:** Correlation coefficient matrix and square roots of AVEs (shown as diagonal elements).

	KQ	KSB	PEOU	PU	RE	TK	TR
KQ	**0.877**						
KSB	0.813	**0.864**					
PEOU	0.765	0.793	**0.898**				
PU	0.685	0.833	0.746	**0.927**			
RE	0.625	0.628	0.562	0.535	**0.873**		
TK	0.797	0.743	0.727	0.686	0.547	**0.827**	
TR	0.829	0.789	0.66	0.659	0.662	0.693	**0.881**

The bold numbers in Table 3 are the square root of AVE.

**Table 4:** Results of a hypothesis test.

Hypothesis	Path	WeChat, *N* = 336			Supported
		Model 1	Model 2	Model 3	
	(Control variables)				
	Gender	−0.025	−0.053	−0.052	
	Age	0.036	0.050	0.037	
	Occupation	−0.018	0.026	0.033	
	Independent variables				
H1	RE- > KSB	0.110^*∗∗*^	0.055	0.050	Partially
H2	TR- > KSB	0.229^*∗∗∗*^	0.212^*∗∗∗*^	0.190^*∗∗∗*^	Yes
H3	KQ- > KSB	0.325^*∗∗∗*^	0.164^*∗∗*^	0.233^*∗∗∗*^	Yes
H4	TK- > KSB	0.214^*∗∗∗*^	0.028	−0.001	Partially
	Moderator				
	PU- > KSB		0.409^*∗∗∗*^	0.415^*∗∗∗*^	
	PEOU- > KSB		0.165^*∗*^	0.143^*∗*^	
H5	PU × RE- > KSB			−0.068	No
H6	PU × TR- > KSB			0.070	No
H7	PU × KQ- > KSB			−0.209^*∗*^	No
H8	PU × TK- > KSB			0.200^*∗*^	Yes
H9	PEOU × RE- > KSB			0.069	No
H10	PEOU × TR- > KSB			−0.099	No
H11	PEOU × KQ- > KSB			0.257^*∗∗*^	Yes
H12	PEOU × TK- > KSB			−0.220^*∗*^	No
R2		0.736	0.837	0.848	

Notes:  ^*∗*^*p*<0.05;  ^*∗∗*^*p*<0.01;  ^*∗∗∗*^*p*<0.001

## Data Availability

The data used to support the findings of this study are included within the article.
